# Supplementary figures and images for: Enhanced in vivo-imaging in medaka by optimized anaesthesia, fluorescent protein selection and removal of pigmentation
Source: PLoS One. 2019 Mar 7;14(3):e0212956. doi: 10.1371/journal.pone.0212956 (PMC6405165; doi:10.1371/journal.pone.0212956)

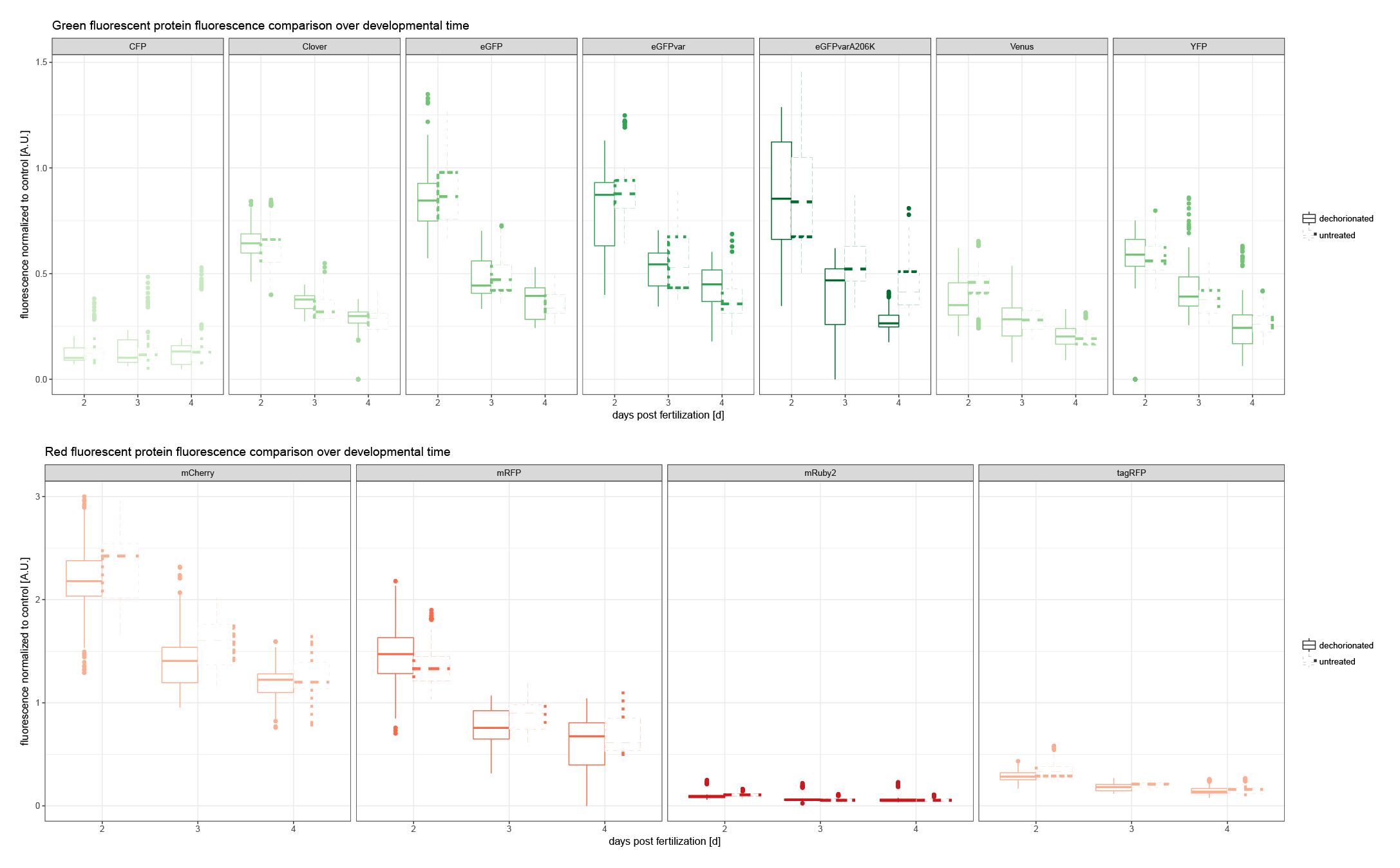

Supplement: S1 Fig — Embryos were injected as previously mentioned. Half of each condition was dechorionated. Both conditions were imaged at 2, 3 and 4dpf and compared to each other. Optical properties of the chorion were negligible and therefore neglected in the analysis since dechorionation has a larger impact on development, especially when performed in an early stage (2–4 dpf). (TIF) [file pone.0212956.s001.tif]

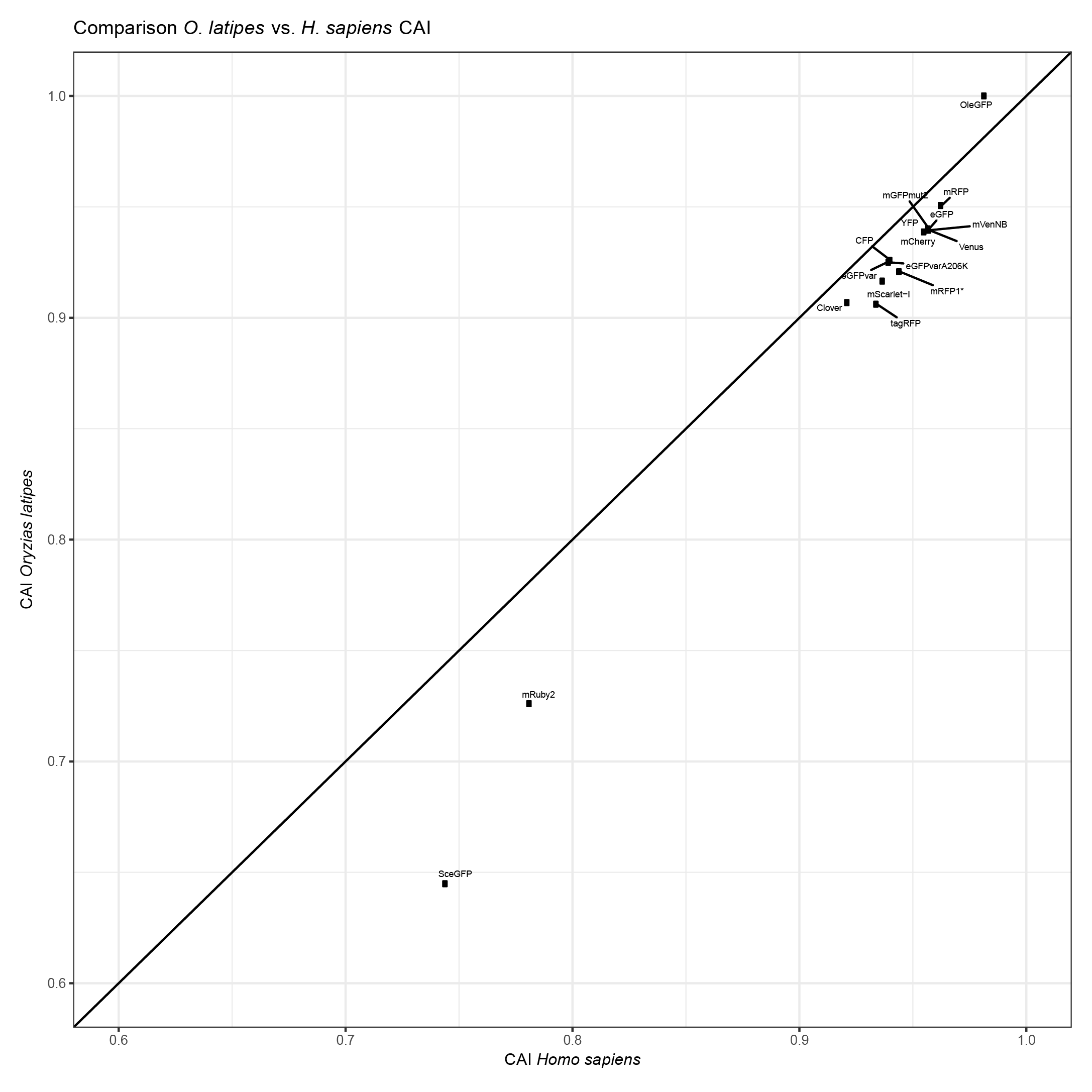

Supplement: S2 Fig — Codon adaptation index of each sequence is plotted for medaka and human along with a diagonal line for orientation. (TIF) [file pone.0212956.s002.tif]

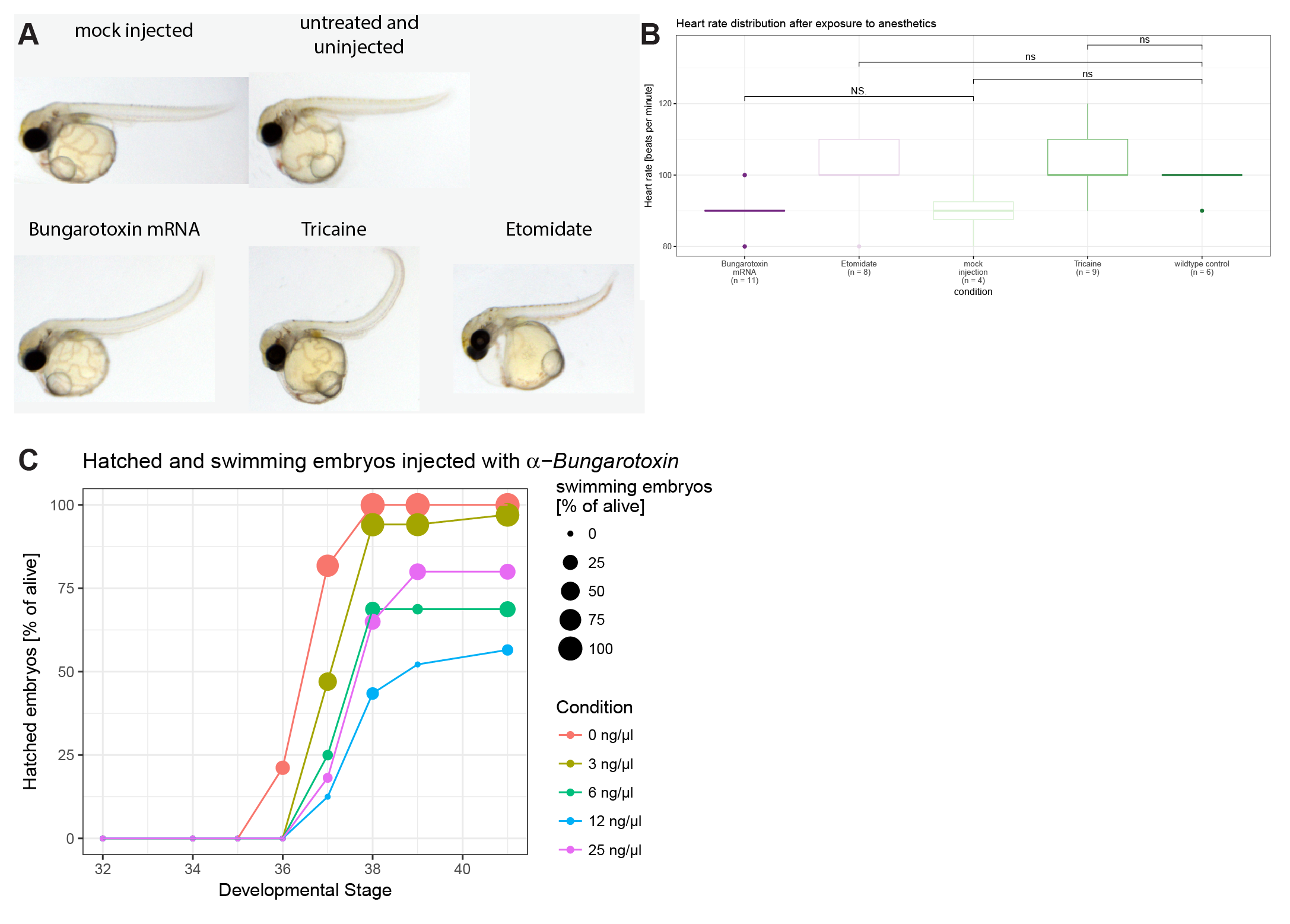

Supplement: S3 Fig — (A) Embryos after demounting from the 96-well plate and finishing the assay (stage 31). No cardiac defects are visible in both controls and embryos injected with α-Bungarotoxin mRNA. For Tricaine, mild defects can be detected, whereas severe defects can be observed in Etomidate treated embryos. (B) Heart rate of α-Bungarotoxin is statistically indistinguishable to its control (mock injection). Mild defects and changes can be observed in Tricaine and Etomidate treated embryos (asterisks indicate P-values: **** P < = 0.0001, *** P < = 0.001, ** P < = 0.01, * P < = 0.05, ns P > 0.05). (C) Different dilutions of α-Bungarotoxin were tested in order to adjust the concentration so fish would wake up after microscopy. Embryos were injected with 0, 3, 6, 12 or 25 ng/μl α-Bungarotoxin mRNA along with eGFP mRNA as an injection control. The number of hatched embryos and the number of swimming embryos per condition was scored (0 ng/μl: n = 33 fish, 3 ng/μl: n = 34 fish, 6 ng/μl: n = 16 fish, 12 ng/μl: n = 24 fish, 25 ng/μl: n = 22 fish). (TIF) [file pone.0212956.s003.tif]

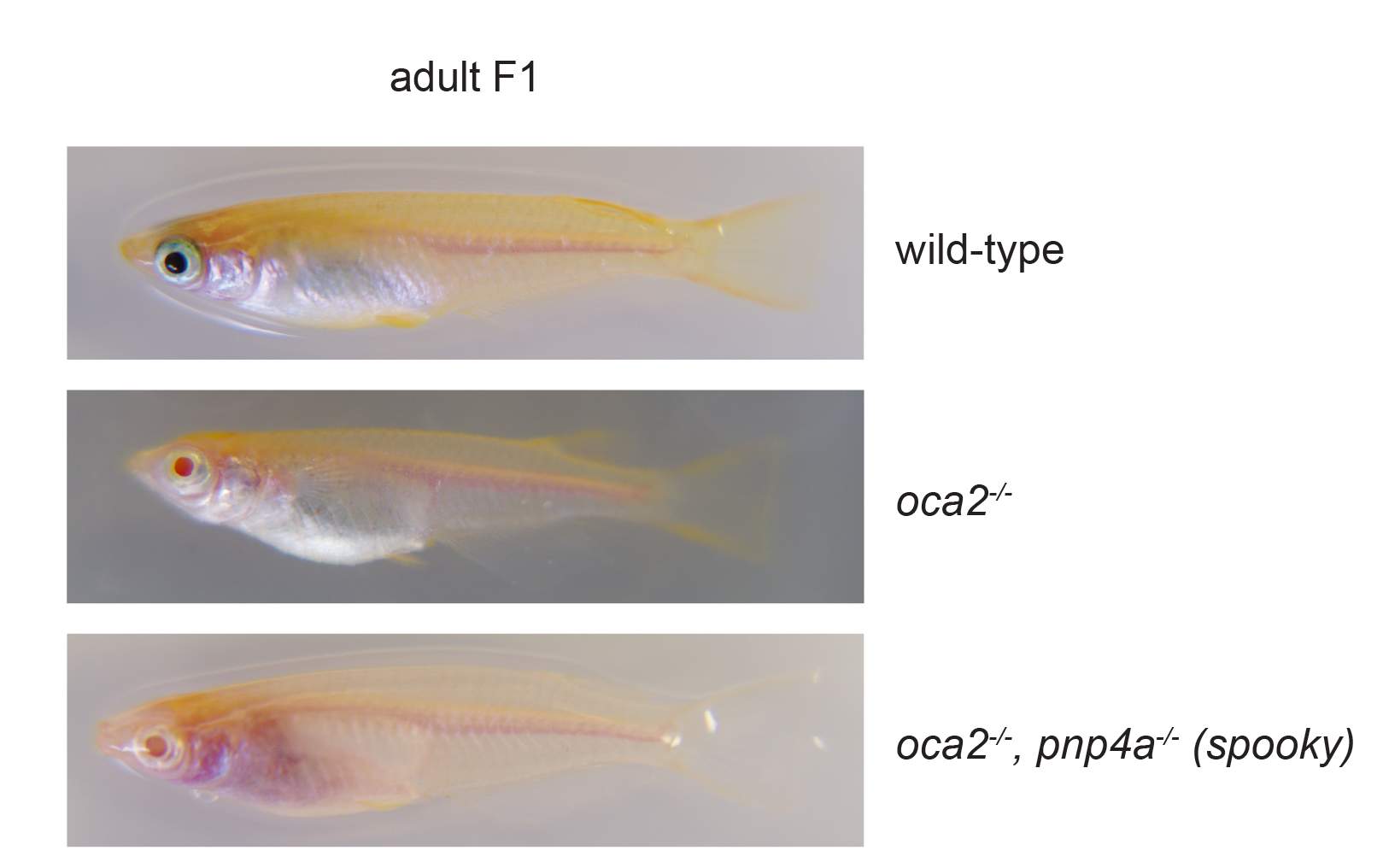

Supplement: S4 Fig — Addition to Fig 5B. Adult fish of the wild-type strain, the oca2-/- mutant line and the double pigment knockout line were imaged. (TIF) [file pone.0212956.s004.tif]
